# Supplementary material for: Primate retina trades single-photon detection for high-fidelity contrast encoding
Source: Nat Commun. 2024 May 27;15:4501. doi: 10.1038/s41467-024-48750-y (PMC11130139; doi:10.1038/s41467-024-48750-y)
Supplement: Supplementary file 1 — Supplementary Information [file 41467_2024_48750_MOESM1_ESM.pdf]

Supplementary information

Primate retina trades single-photon detection for high-fidelity contrast encoding

Markku Kilpeläinen, Johan Westö, Jussi Tiihonen, Anton Laihi, Daisuke Takeshita, Fred Rieke & Petri Ala-Laurila

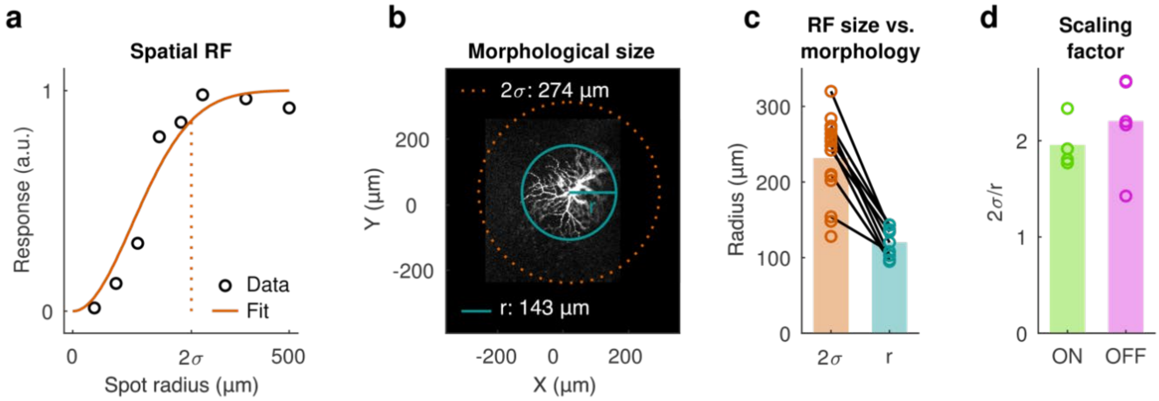

Supplementary Fig. 1. The size of the spatial RF is larger than the width of the dendritic tree in darkness. a, The size of the spatial RF was measured by fitting a Gaussian-shaped RF to the responses obtained while presenting 250 ms dim spots of various sizes. **b**, The size of the dendritic tree was measured by fitting a circle to the edges of the dendritic tree. **c**, The size of the RF (defined as 2σ) was always larger than the dendritic tree (lines denote measurements from the same cell). **d**, The difference could be explained with a scaling factor ($\frac{2\sigma}{r}$) of roughly 2 for both ON and OFF parasols.

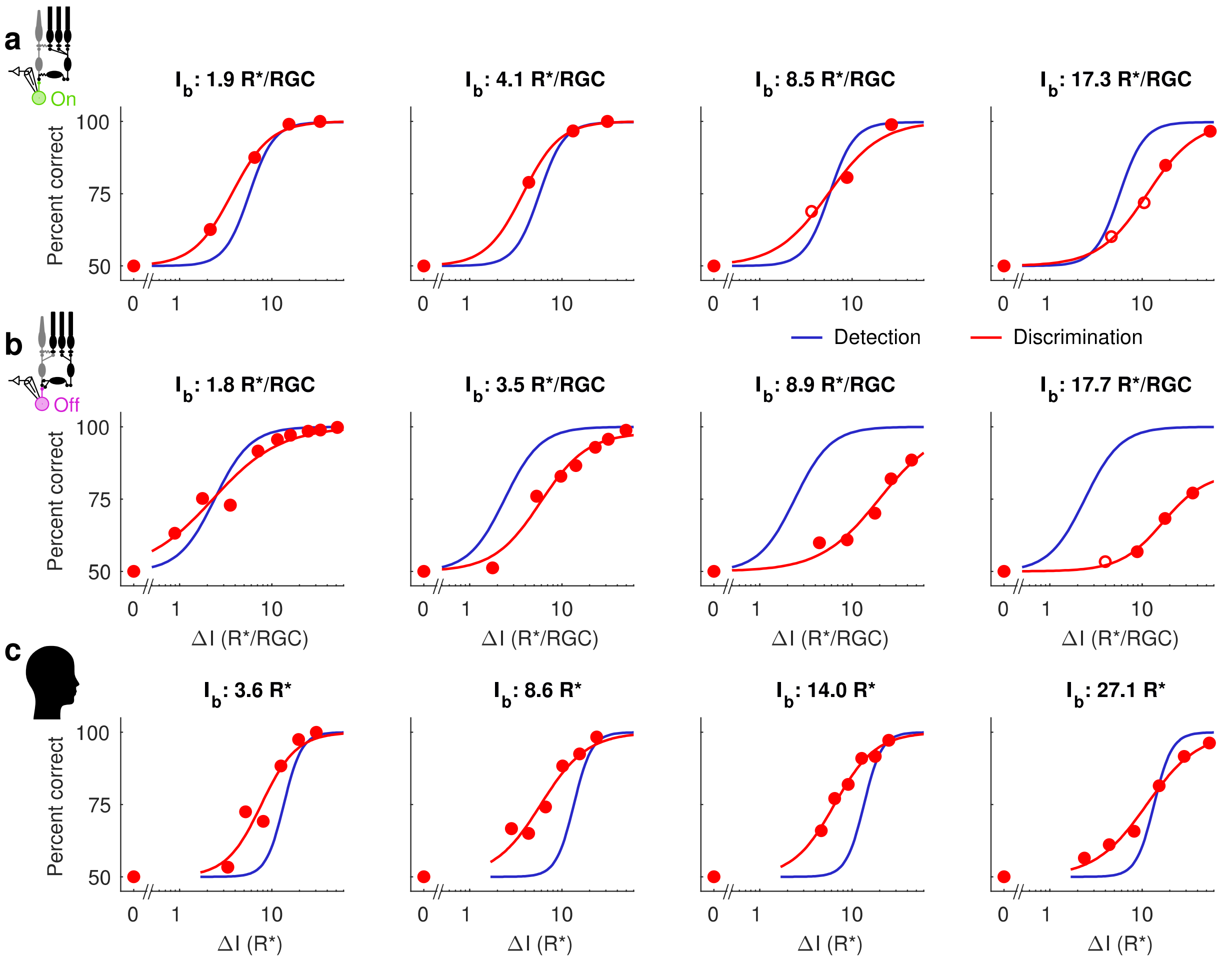

Supplementary Fig. 2. 2AFC curves were fitted to all reference intensities and additional response distributions were modelled if needed. a, 2AFC curves for four different reference intensities for an example ON RGC. Filled red circles correspond to data points obtained from measured response distributions and open circles correspond to data points obtained from modelled distributions. The red and blue lines denote the fitted Hill function for the discrimination and the detection task, respectively. **b**, Same as **a**, but for an example OFF RGC. **c**, Same as **a**, but for one psychophysical observer (O1).

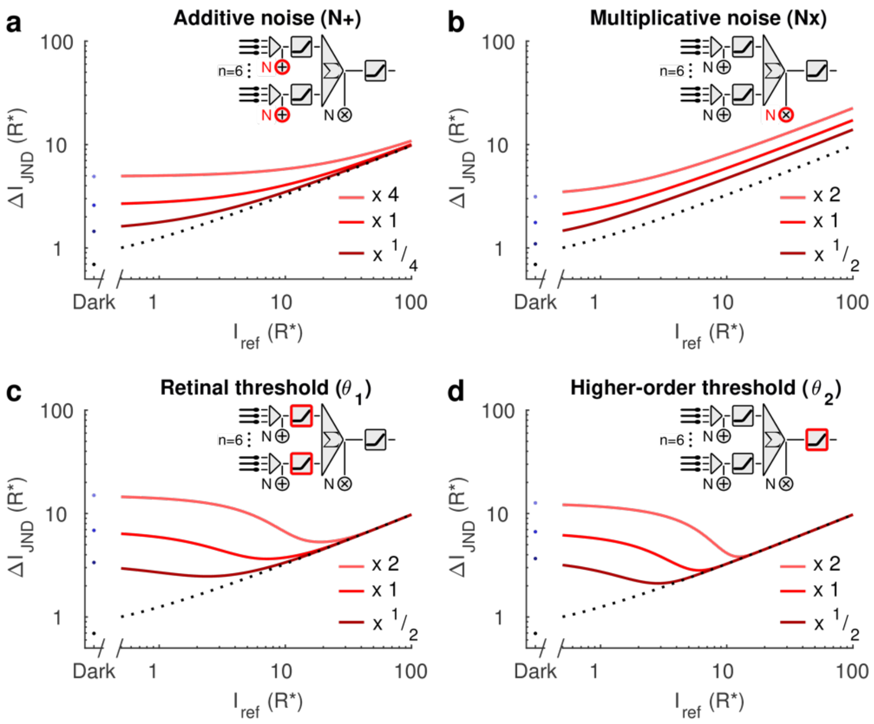

Supplementary Fig. 3. Effects of varying each model parameter separately with respect to the ideal noiseless case without thresholds (dotted black line). a, Additive Poisson noise mainly degrades the model's performance for low reference intensities, and this effect vanishes for larger reference intensities. **b**, Multiplicative noise degrades the model's performance over all reference intensities. **c** and **d**, Thresholding nonlinearites result in clear dips that increase in depth as the threshold increases. In each panel, the parameters were either scaled up or down by a constant factor (2 or 4). The base parameter values (1 X) for the model were the following: Additive noise = 1 R*/subunit, Multiplicative noise = 0.5, Retinal threshold (θ_1) = 2 R*/subunit, Higher-order threshold (θ_2) = 6 R*. The model assumes 6 subunits (n = 6) and 2000 rods per subunit.

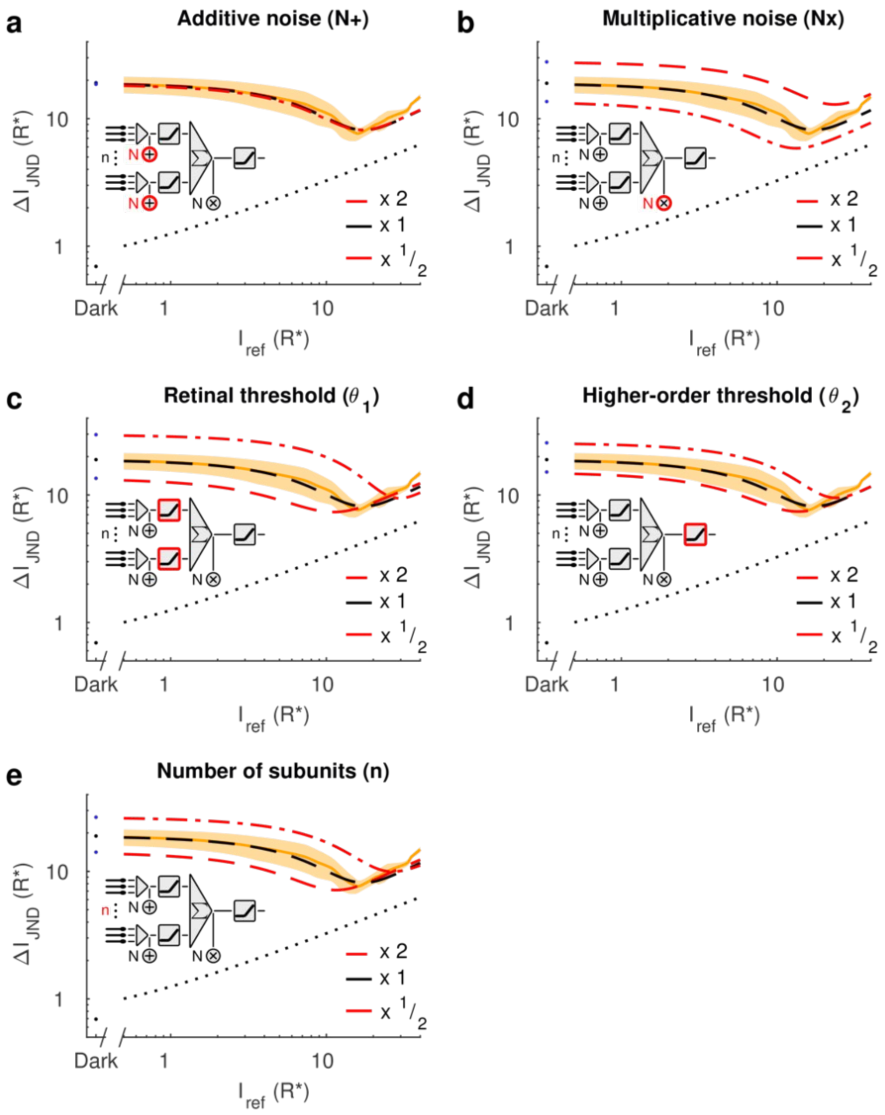

Supplementary Fig. 4. Robustness analysis for model parameters. a-e, Each model parameter is halved or doubled one at a time around the fitted values found for M2 (Figure 3e). **a** Additive noise (N+), **b** Multiplicative noise (Nx), **c** Retinal threshold (θ_1), **d** Higher-order threshold (θ_2), **e** Number of subunits (*n*). The analysis is performed around the values: 0.055 (additive noise), 0.333 (multiplicative noise), 2 (retinal threshold), 2 (higher-order threshold), 6 (number of subunits).
