# Supplementary material for: Primate retina trades single-photon detection for high-fidelity contrast encoding
Source: Nat Commun. 2024 May 27;15:4501. doi: 10.1038/s41467-024-48750-y (PMC11130139; doi:10.1038/s41467-024-48750-y)
Supplement: Supplementary file 3 — Reporting Summary [file 41467_2024_48750_MOESM3_ESM.pdf]

nature portfolio

Corresponding author(s): Petri Ala-Laurila

Last updated by author(s): May 2, 2024

Reporting Summary

Nature Portfolio wishes to improve the reproducibility of the work that we publish. This form provides structure for consistency and transparency in reporting. For further information on Nature Portfolio policies, see our Editorial Policies and the Editorial Policy Checklist.

Statistics

For all statistical analyses, confirm that the following items are present in the figure legend, table legend, main text, or Methods section.

n/a	Confirmed	
☐	☒	The exact sample size (n) for each experimental group/condition, given as a discrete number and unit of measurement
☐	☒	A statement on whether measurements were taken from distinct samples or whether the same sample was measured repeatedly
☐	☒	The statistical test(s) used AND whether they are one- or two-sided<br>*Only common tests should be described solely by name; describe more complex techniques in the Methods section.*
☐	☒	A description of all covariates tested
☐	☒	A description of any assumptions or corrections, such as tests of normality and adjustment for multiple comparisons
☐	☒	A full description of the statistical parameters including central tendency (e.g. means) or other basic estimates (e.g. regression coefficient) AND variation (e.g. standard deviation) or associated estimates of uncertainty (e.g. confidence intervals)
☐	☒	For null hypothesis testing, the test statistic (e.g. F, t, r) with confidence intervals, effect sizes, degrees of freedom and P value noted<br>*Give P values as exact values whenever suitable.*
☒	☐	For Bayesian analysis, information on the choice of priors and Markov chain Monte Carlo settings
☒	☐	For hierarchical and complex designs, identification of the appropriate level for tests and full reporting of outcomes
☐	☒	Estimates of effect sizes (e.g. Cohen's d, Pearson's r), indicating how they were calculated

Our web collection on statistics for biologists contains articles on many of the points above.

Software and code

Policy information about availability of computer code

Data collection	Human psychophysics: Data acquisition and stimulus delivery was controlled using MATLAB (version: R2013a) and the Data Acquisition Toolbox.<br>Primate RGC electrophysiology: Data were acquired with MATLAB (version: R2013a) and the Symphony data acquisitions system (https://symphony-das.github.io/) using a Multiclamp 700B amplifier and ITC-18 A/D board. Visual stimuli were generated using the Stage software package (https://stage-vss.github.io/).
Data analysis	All analyses and models were performed in MATLAB (version: R2017b). The code is custom made for this manuscript, but parts of the analyses largely rely on upon previously published methods, such as the 2AFC ideal observer analyses (Ala-Laurila & Rieke, 2014). For the code, see the zenodo repository: http://dx.doi.org/10.5281/zenodo.10459936.

For manuscripts utilizing custom algorithms or software that are central to the research but not yet described in published literature, software must be made available to editors and reviewers. We strongly encourage code deposition in a community repository (e.g. GitHub). See the Nature Portfolio guidelines for submitting code & software for further information.

Data

Policy information about availability of data

All manuscripts must include a data availability statement. This statement should provide the following information, where applicable:

- Accession codes, unique identifiers, or web links for publicly available datasets
- A description of any restrictions on data availability
- For clinical datasets or third party data, please ensure that the statement adheres to our policy

Source data are provided with this paper. All data generated and analyzed in this study are available in the Figshare database: https://doi.org/10.6084/m9.figshare.25737099.

Research involving human participants, their data, or biological material

Policy information about studies with human participants or human data. See also policy information about sex, gender (identity/presentation), and sexual orientation and race, ethnicity and racism.

Reporting on sex and gender	Findings apply to both sexes. Sex was considered in the study design to the extent that both (self-reported) female and male participants were recruited. Sex was not a variable of interest in the study. However, all analyses were made on individual participant level, and results are very similar for male and female observers. Sample size does not warrant a statistical test. Data is presented on individual participant level. We state in methods which participants were female.
Reporting on race, ethnicity, or other socially relevant groupings	No categorization was made based on any socially relevant groupings.
Population characteristics	Age 19–29 years. No covariates were used as each observer was analyzed on single observer level.
Recruitment	-O1 was an author and member of the research group.<br>-O2 and O5 were previously unacquainted students who volunteered, when study information was disseminated to students in their break room.<br>-O3 was a (19 year old) high school student who volunteered, when study information was disseminated in a scout meeting.<br>-O4 was a member of the research group, but naive to the purposes of the study<br><br>Potentially, people who consider themselves to see poorly in the dark would be more likely to decline from participating. However, this is a study of the normally functioning human visual system, not a population study. We point out that the results of all 5 observers are quite similar. If individuals with night vision difficulties (who were still able to carry out the task) were included, all detection and discrimination thresholds would be expected to be higher. However, the characteristic shape of the dipper functions would be expected to stay the same (see e.g., Bradley & Ohzawa, 1986, Vision Research)
Ethics oversight	University of Helsinki Ethical review board in humanities and social and behavioral sciences approved the protocol

Note that full information on the approval of the study protocol must also be provided in the manuscript.

Field-specific reporting

Please select the one below that is the best fit for your research. If you are not sure, read the appropriate sections before making your selection.

☒ Life sciences ☐ Behavioural & social sciences ☐ Ecological, evolutionary & environmental sciences

For a reference copy of the document with all sections, see nature.com/documents/nr-reporting-summary-flat.pdf

Life sciences study design

All studies must disclose on these points even when the disclosure is negative.

Sample size	Human psychophysics: The study followed the small n design (Smith & Little, 2018, Psychonomic Bulletin and Review), where an extensive amount of data is collected for each individual observer, and the main results are significant on the level of each individual observer. Thus, n = 5 is based only on the need to show that our results are not based on one or two abnormal observers.<br>Primate RGC electrophysiology: The dataset included data from 54 ON parasol RGCs and 11 OFF parasol RGCs. No statistical methods were used to determine the sample size. Based on earlier, similar RGC experiments (Ala-Laurila & Rieke, 2014) we have shown that the significant differences in the response characteristics between ON and OFF parasol RGCs can be reliably quantified in the sample sizes that are similar to the ones used in this study. The narrow SEMs of both our ON and OFF RGC data (see Fig. 3d) indicate that the sample size is sufficient.
Data exclusions	Human psychophysics: No data was excluded.<br>Primate RGC electrophysiology: Ganglion cells were included in the main analyses if they filled similar sensitivity criteria as established earlier by Ala-Laurila & Rieke (2014): RGCs had to generate a response that differed form the baseline firing rate by four to five spikes on average when stimulated with a brief flash producing 0.001–0.002 R*/rod.

Replication	Human psychophysics: The experiment was replicated in total 5 times using 5 independent observers all together. Each indented observer represents on independent replication of the experiment. The fact that the data from all observers produces very similar results (and the fact that similar results have earlier been found with slightly different experimental parameters), suggests, that the results are likely to be highly replicable.<br>Primate RGC electrophysiology: RGC sensitivities in darkness are in line with results from previous studies and thus replicable (Ala-Laurila & Rieke, 2014)
Randomization	Human psychophysics: The study included only within-subjects comparisons. There are no comparisons between groups. Thus, the impact of covariates is minimized as the detection and discrimination performance is compared within each subject.<br>Primate electrophysiology: no randomisation. Covariates were not included, as we have not observed any systematic changes in ON and OFF parasol RGC response properties related to non-experimental parameters (including the age and gender of the animal where the retina was harvested from). Particularly, we have observed that the major variance component of the response properties of parasol RGCs is related to harvesting the dark-adapted ex vivo retinas at the uttermost sensitive conditions. We used similar criteria as previously (Ala-Laurila & Rieke, 2014) for validating the conditions of the ex vivo retinal preparations.
Blinding	Human experiments: There was no group allocation.<br>Primate RGC electrophysiology: Classification ON and OFF parasol ganglion cells during the recordings is based on the morphology of these cells as well as their response properties as previously reported (Ala-Laurila & Rieke, 2014). Thus, the experimentalist has direct knowledge of the cell identity during the recording and blinding was not possible. Similar light stimuli were delivered to both ON and OFF parasol RGCs and the data analysis applied post recordings was also similar for both cell types. Thus, blinding should not impact the final results obtained.

Reporting for specific materials, systems and methods

We require information from authors about some types of materials, experimental systems and methods used in many studies. Here, indicate whether each material, system or method listed is relevant to your study. If you are not sure if a list item applies to your research, read the appropriate section before selecting a response.

Materials & experimental systems

n/a	Involved in the study
☒	☐ Antibodies
☒	☐ Eukaryotic cell lines
☒	☐ Palaeontology and archaeology
☐	☒ Animals and other organisms
☒	☐ Clinical data
☒	☐ Dual use research of concern
☒	☐ Plants

Methods

n/a	Involved in the study
☒	☐ ChIP-seq
☒	☐ Flow cytometry
☒	☐ MRI-based neuroimaging

Animals and other research organisms

Policy information about studies involving animals; ARRIVE guidelines recommended for reporting animal research, and Sex and Gender in Research

Laboratory animals	Studies were performed with tissues obtained from male and female macaque monkeys (Macaca fascicularis, Macaca mulatta and Macaca nemestrina) obtained through the Tissue Distribution Program of the National Primate Research Center at the University of Washington. Animals ranged in age from 4-21 years.
Wild animals	This study did not involve wild animals.
Reporting on sex	Findings apply to both sexes. Sex was considered in the study design to the extent that tissue was acquired from both female and male animals. Sex was not a variable of interest in the study. The study design did not include testing the effect of sex and the sample is not suited for a formal test. There were no observable differences between RGC cells from female and male animals.
Field-collected samples	This study did not involve collection of samples from the field.
Ethics oversight	All procedures were approved by the University of Washington Institutional Animal Care and Use Committee.

Note that full information on the approval of the study protocol must also be provided in the manuscript.
